# Supplementary material for: Novel Anti-Aging Benzoquinone Derivatives from Onosma bracteatum Wall
Source: Molecules. 2019 Apr 11;24(7):1428. doi: 10.3390/molecules24071428 (PMC6479397; doi:10.3390/molecules24071428)
Supplement: Supplementary file 1 [file molecules-24-01428-s001.pdf]

Supplementary Data for

Novel Anti-Aging Benzoquinone Derivatives from *Onosma bracteatum* Wall

Umer Farooq [1], Yanjun Pan [1], Dejene Disasa [1] and Jianhua Qi [1,*]

[1] College of Pharmaceutical Sciences, Zhejiang University, Yu Hang Tang Road 866, Hangzhou 310058, China; 11519039@zju.edu.cn (U.F.); 21719029@zju.edu.cn (Y.P.); 11719053@zju.edu.cn (D.D.)

* Correspondence: qijianhua@zju.edu.cn; Tel.: +86-571-8820-8627 (J.Q.)

Contents

Figure S1. ^{1}H NMR spectrum of allomicrophyllone (**1**) measured in $CDCl_3$.

Figure S2. ^{13}C NMR spectrum of allomicrophyllone (**1**) measured in $CDCl_3$.

Figure S3. ^{1}H NMR spectrum of ehretiquinone (**2**) measured in $CDCl_3$.

Figure S4. ^{13}C NMR spectrum of ehretiquinone (**2**) measured in $CDCl_3$.

Figure S5. ^{1}H NMR spectrum of ehretiquinone B (**3**) measured in $CDCl_3$.

Figure S6. ^{13}C NMR spectrum of ehretiquinone B (**3**) measured in $CDCl_3$.

Figure S7. ^{1}H-^{1}H COSY spectrum of ehretiquinone B (**3**) measured in $CDCl_3$.

Figure S8. HSQC spectrum of ehretiquinone B (**3**) measured in $CDCl_3$.

Figure S9. HMBC spectrum of ehretiquinone B (**3**) measured in $CDCl_3$.

Figure S10. NOESY spectrum of ehretiquinone B (**3**) measured in $CDCl_3$.

Figure S11. ^{1}H NMR spectrum of ehretiquinone C (**4**) measured in $CDCl_3$.

Figure S12. ^{13}C NMR spectrum of ehretiquinone C (**4**) measured in $CDCl_3$.

Figure S13. ^{1}H-^{1}H COSY spectrum of ehretiquinone C (**4**) measured in $CDCl_3$.

Figure S14. HSQC spectrum of ehretiquinone C (**4**) measured in $CDCl_3$.

Figure S15. HMBC spectrum of ehretiquinone C (**4**) measured in $CDCl_3$.

Figure S16. NOESY spectrum of ehretiquinone C (**4**) measured in $CDCl_3$.

Figure S17. ^{1}H NMR spectrum of ehretiquinone D (**5**) measured in $CDCl_3$.

Figure S18. ^{13}C NMR spectrum of ehretiquinone D (**5**) measured in $CDCl_3$.

Figure S19. ^{1}H-^{1}H COSY spectrum of ehretiquinone D (**5**) measured in $CDCl_3$.

Figure S20. HSQC spectrum of ehretiquinone D (**5**) measured in $CDCl_3$.

Figure S21. HMBC spectrum of ehretiquinone D (**5**) measured in $CDCl_3$.

Figure S22. NOESY spectrum of ehretiquinone D (**5**) measured in $CDCl_3$.

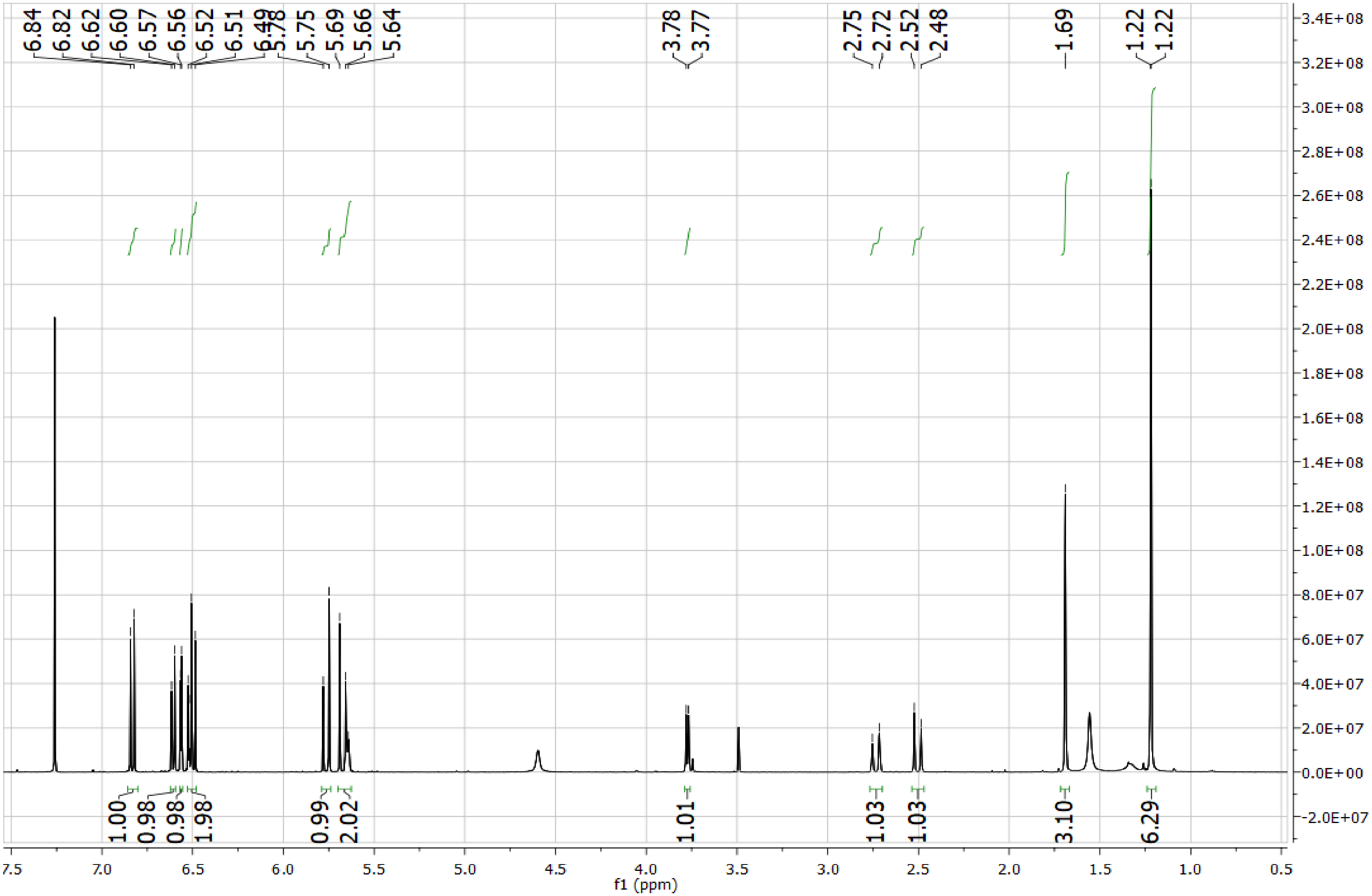

Figure S1. ^{1}H NMR spectrum of allomicrophyllone (**1**) measured in $CDCl_3$.

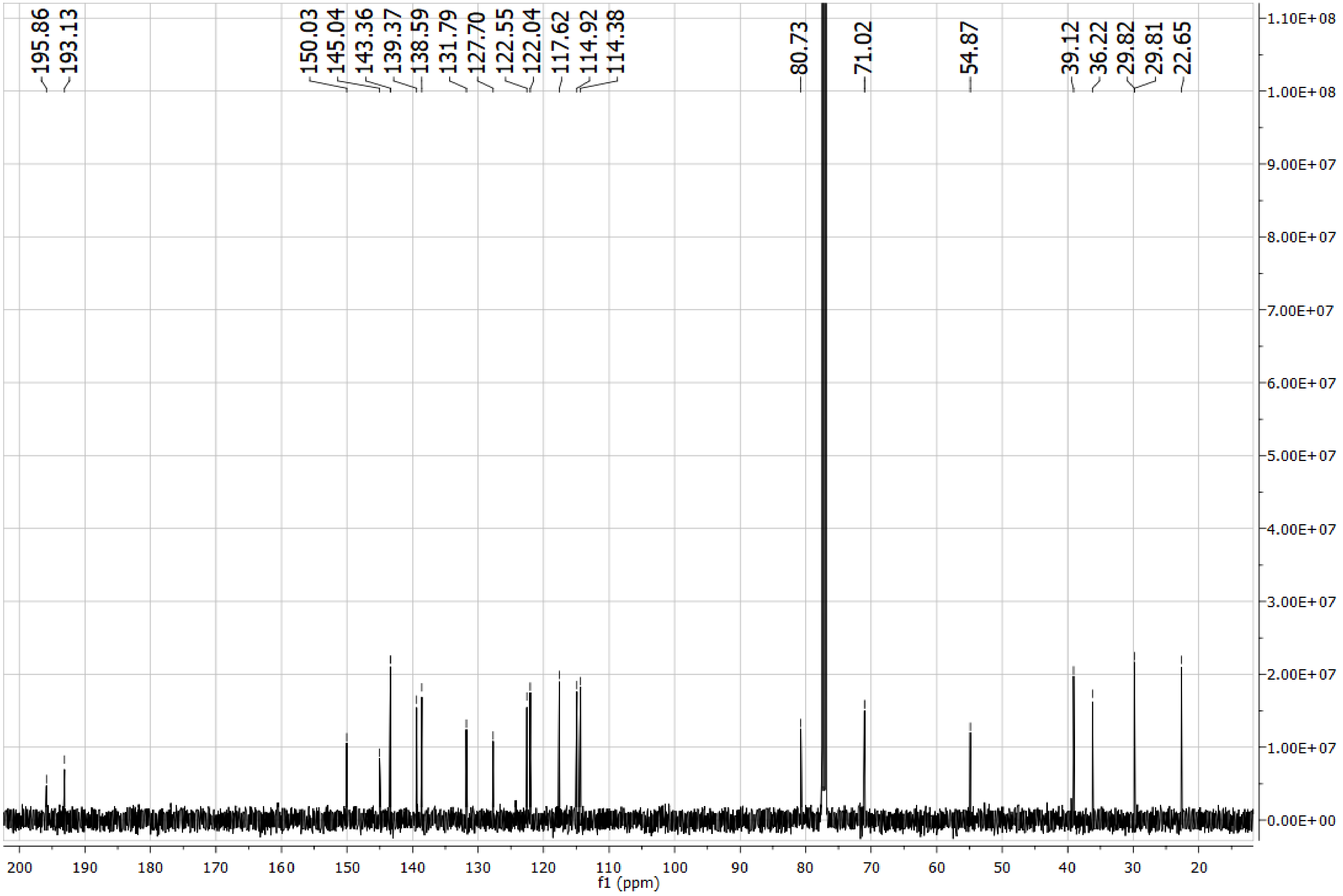

Figure S2. ^{13}C NMR spectrum of allomicrophyllone (**1**) measured in $CDCl_3$.

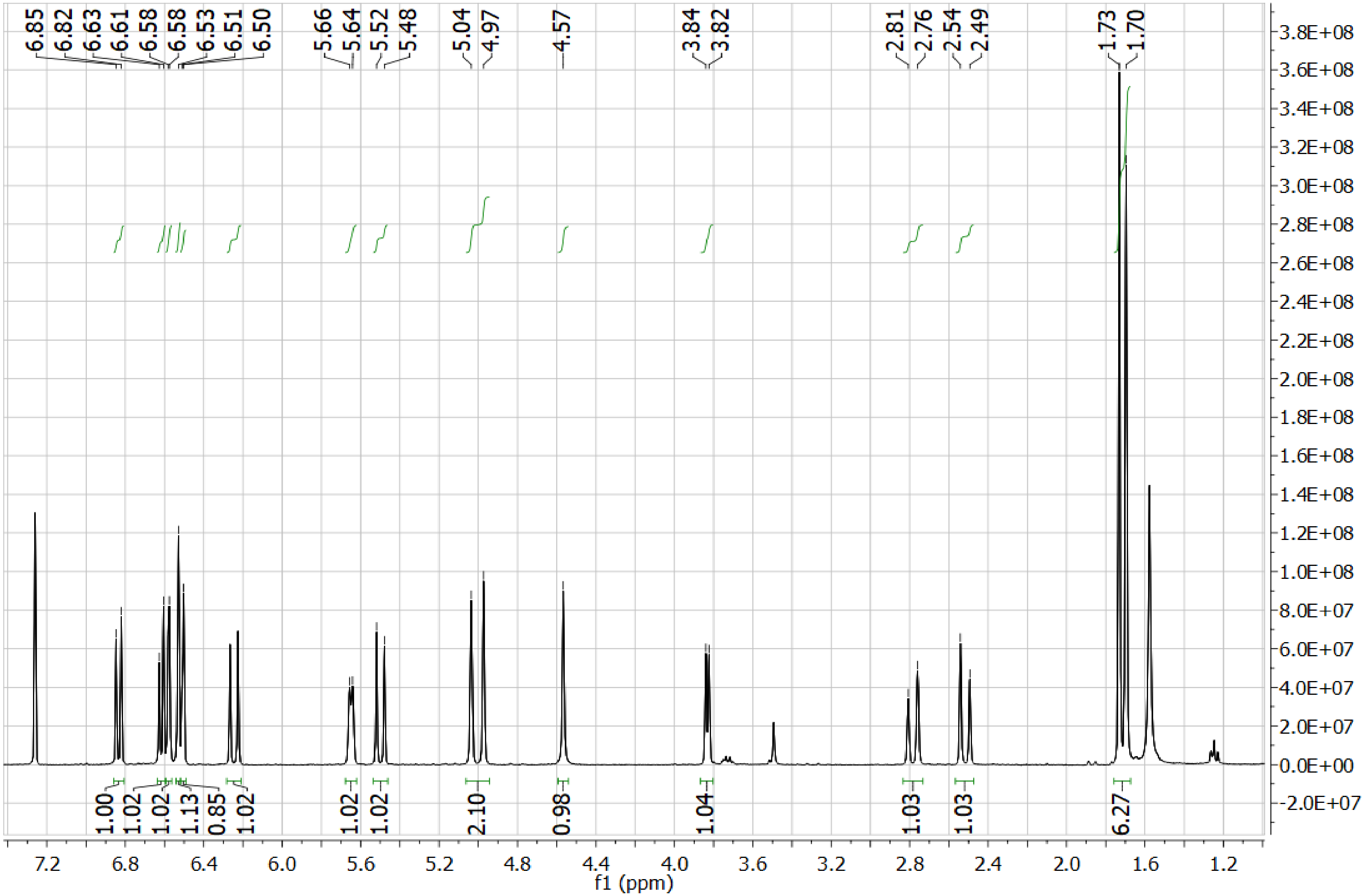

Figure S3. ^{1}H NMR spectrum of ehretiquinone (**2**) measured in $CDCl_3$.

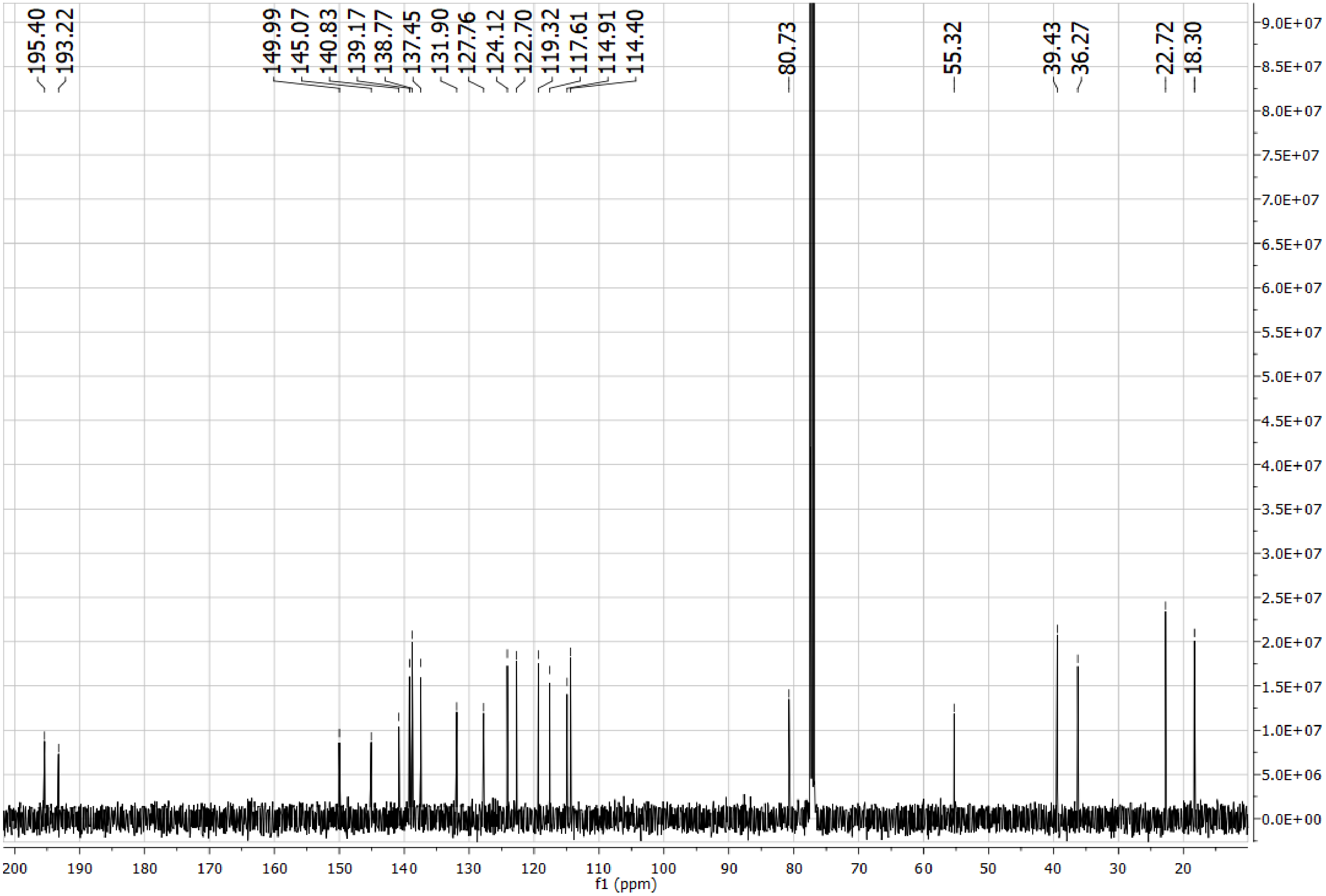

Figure S4. ^{13}C NMR spectrum of ehretiquinone (**2**) measured in $CDCl_3$.

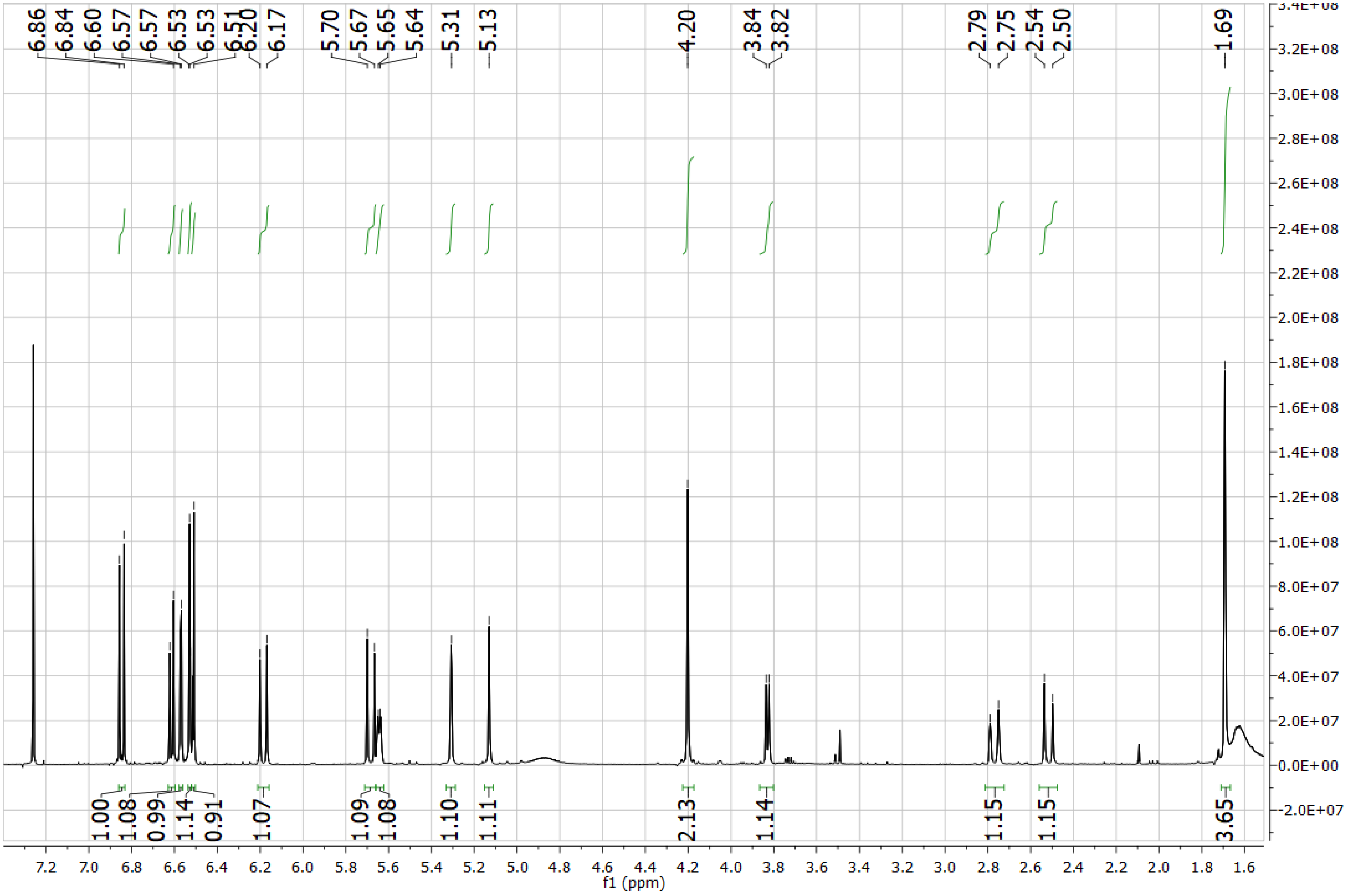

Figure S5. ^{1}H NMR spectrum of ehretiquinone B (**3**) measured in $CDCl_3$.

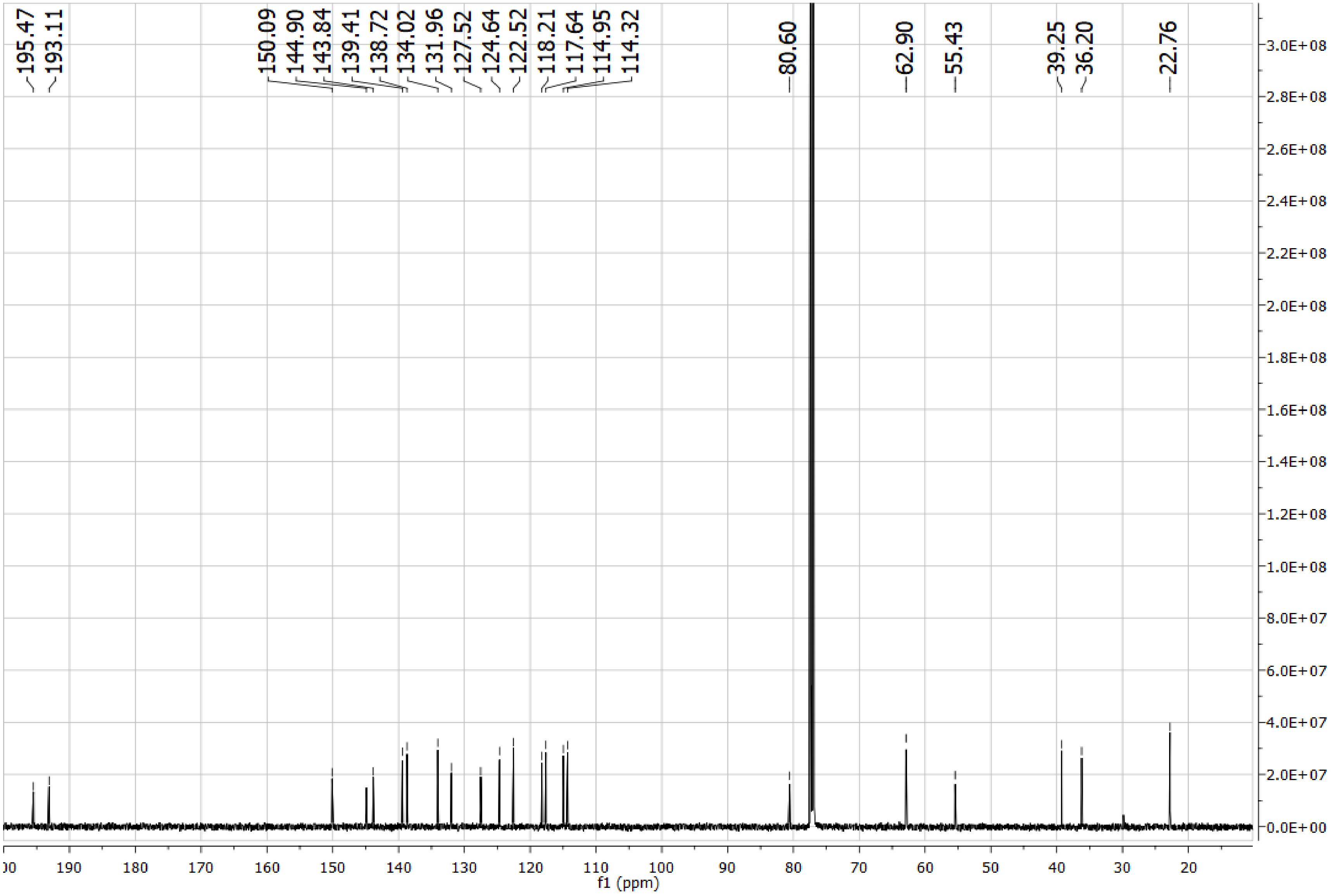

Figure S6. ^{13}C NMR spectrum of ehretiquinone B (**3**) measured in $CDCl_3$.

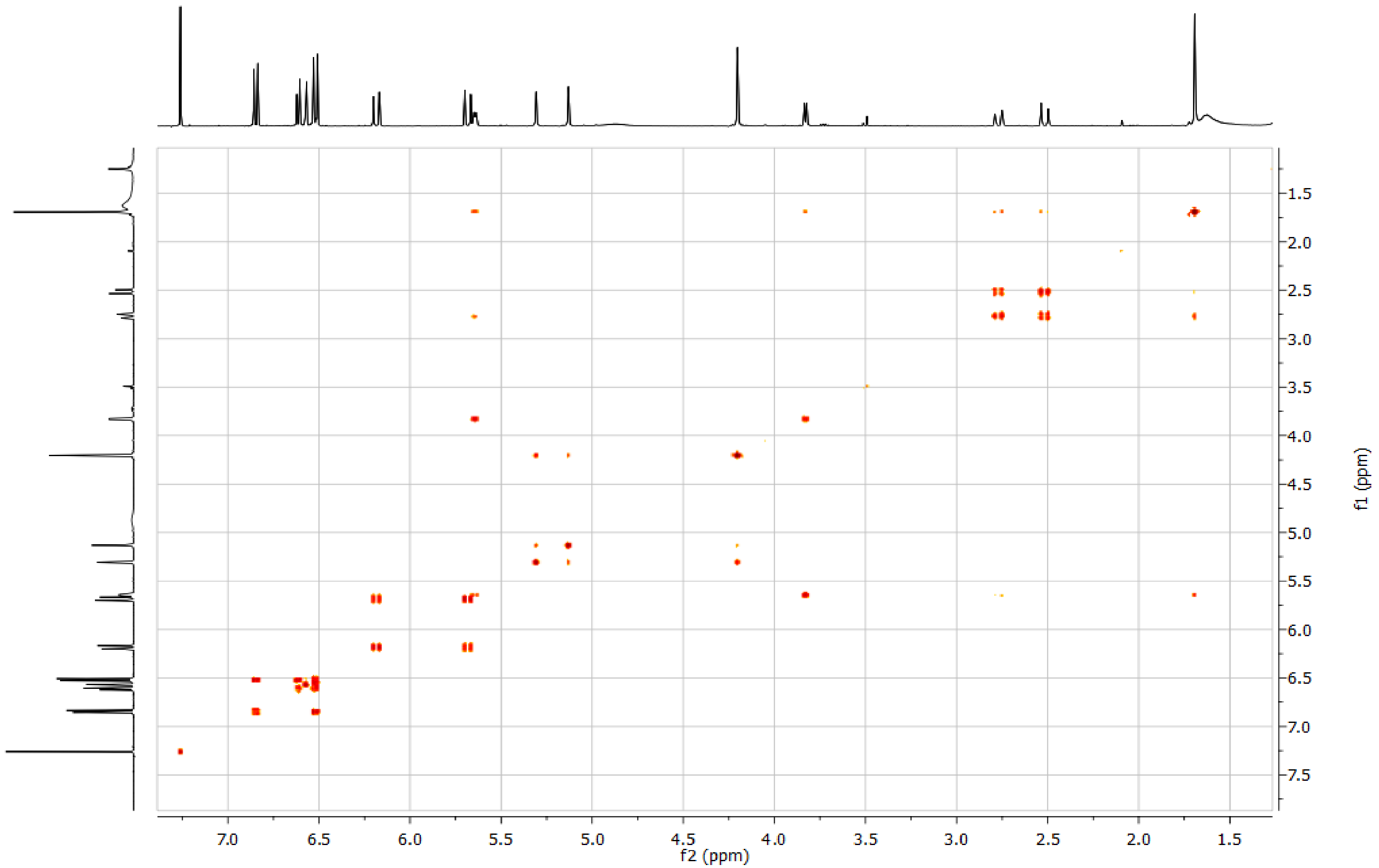

Figure S7. ^{1}H-^{1}H COSY spectrum of ehretiquinone B (**3**) measured in $CDCl_3$.

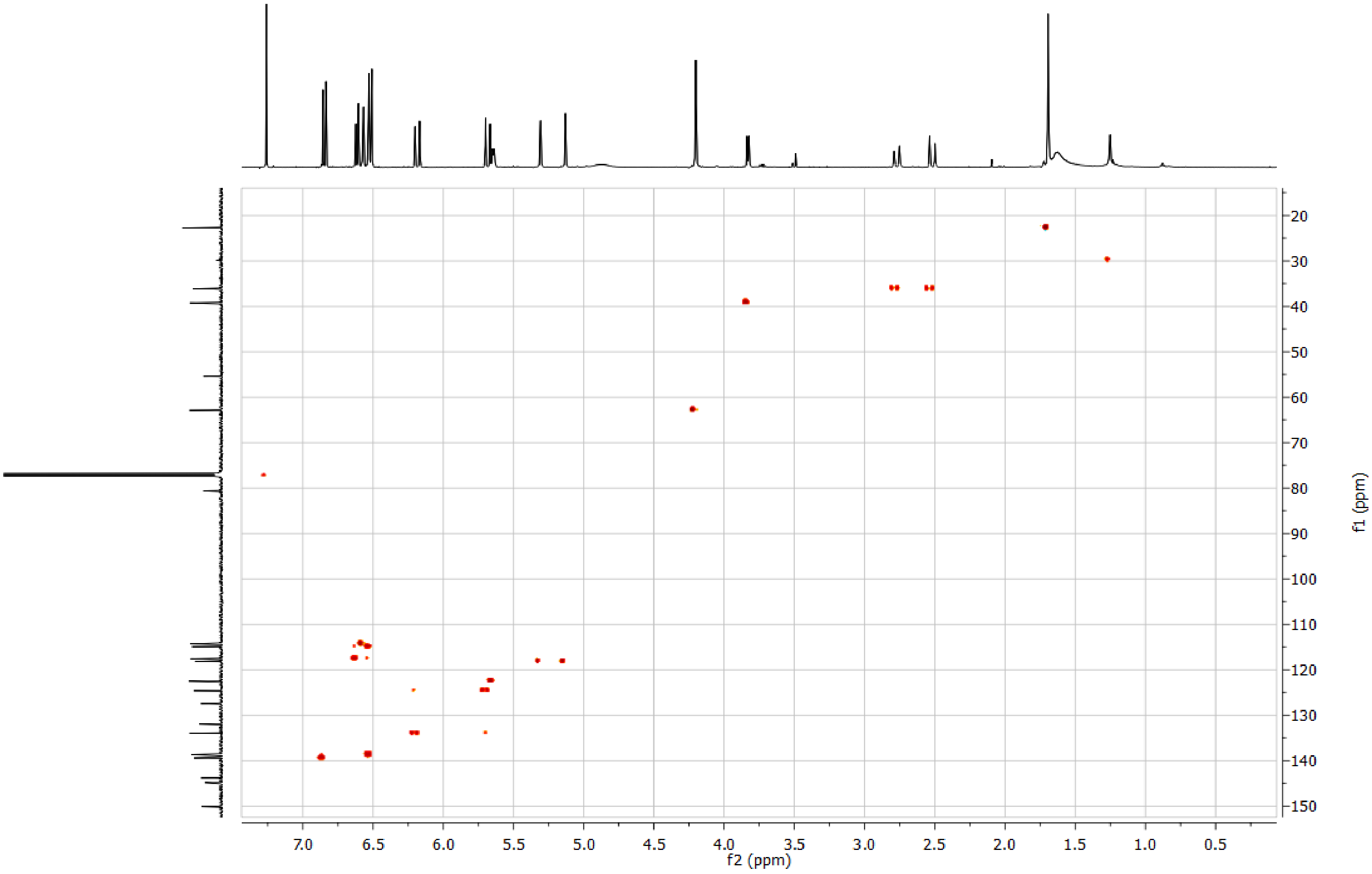

Figure S8. HSQC spectrum of ehretiquinone B (**3**) measured in $CDCl_3$.

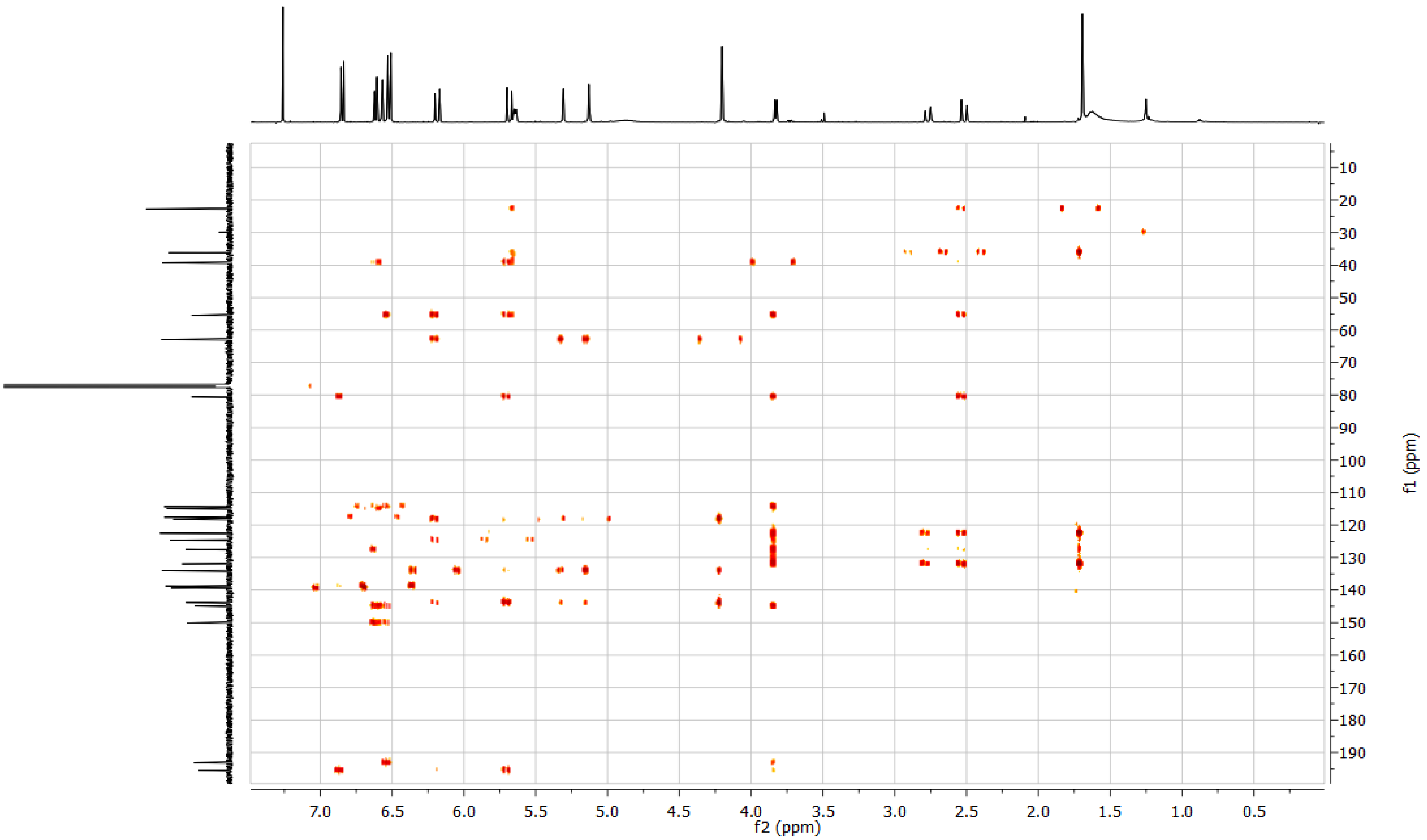

Figure S9. HMBC spectrum of ehretiquinone B (**3**) measured in $CDCl_3$.

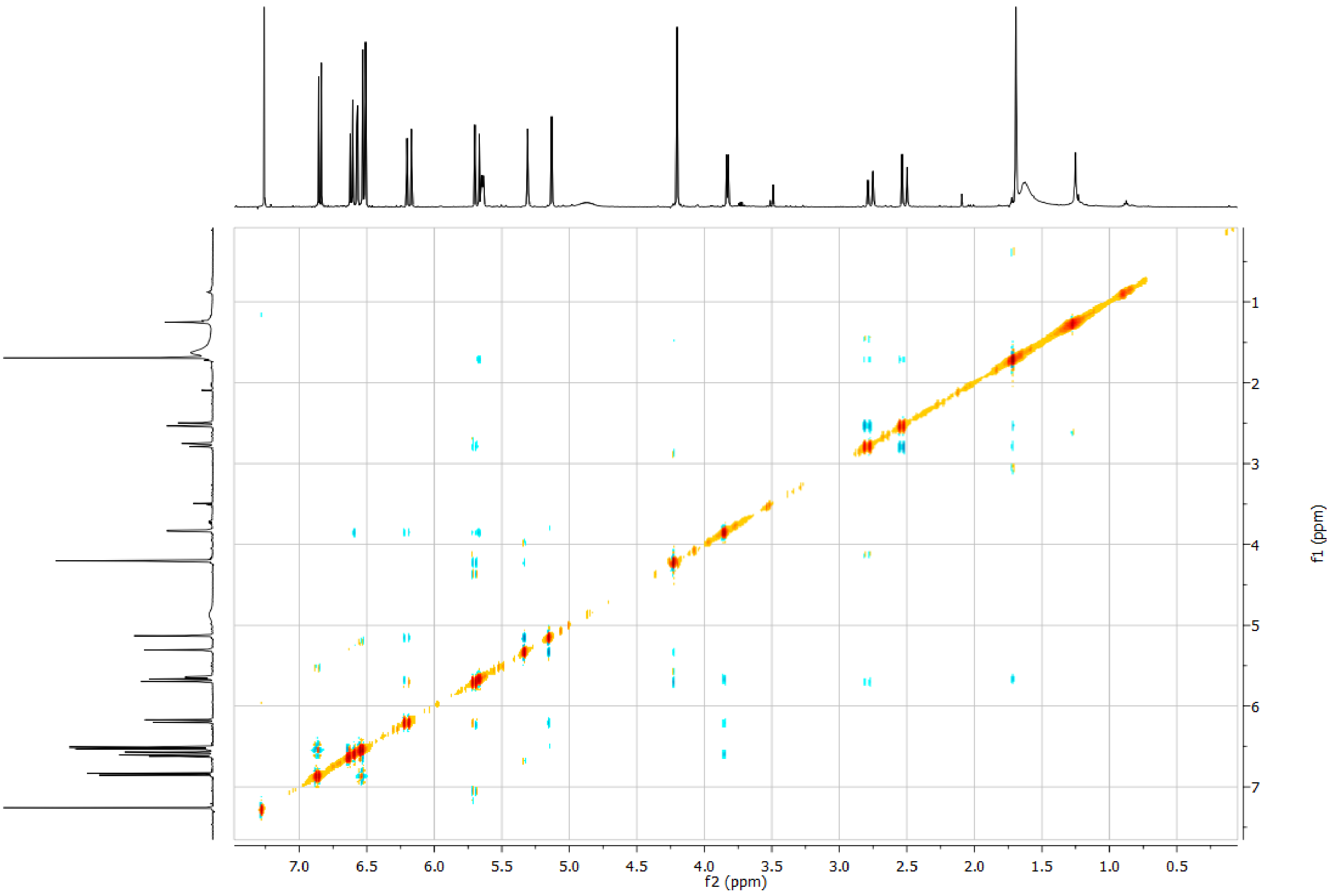

Figure S10. NOESY spectrum of ehretiquinone B (**3**) measured in $CDCl_3$.

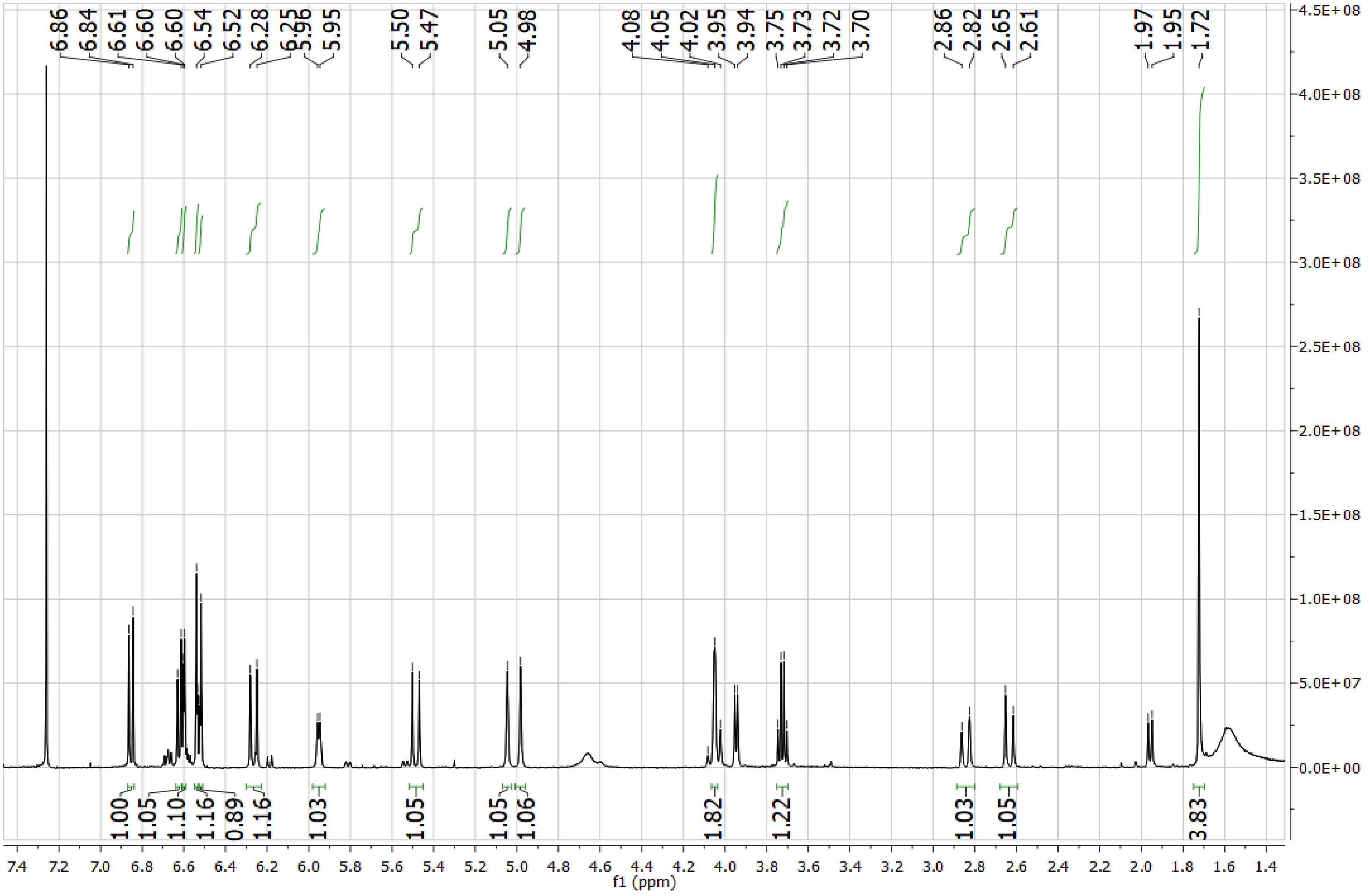

Figure S11. ^{1}H NMR spectrum of ehretiquinone C (**4**) measured in $CDCl_3$.

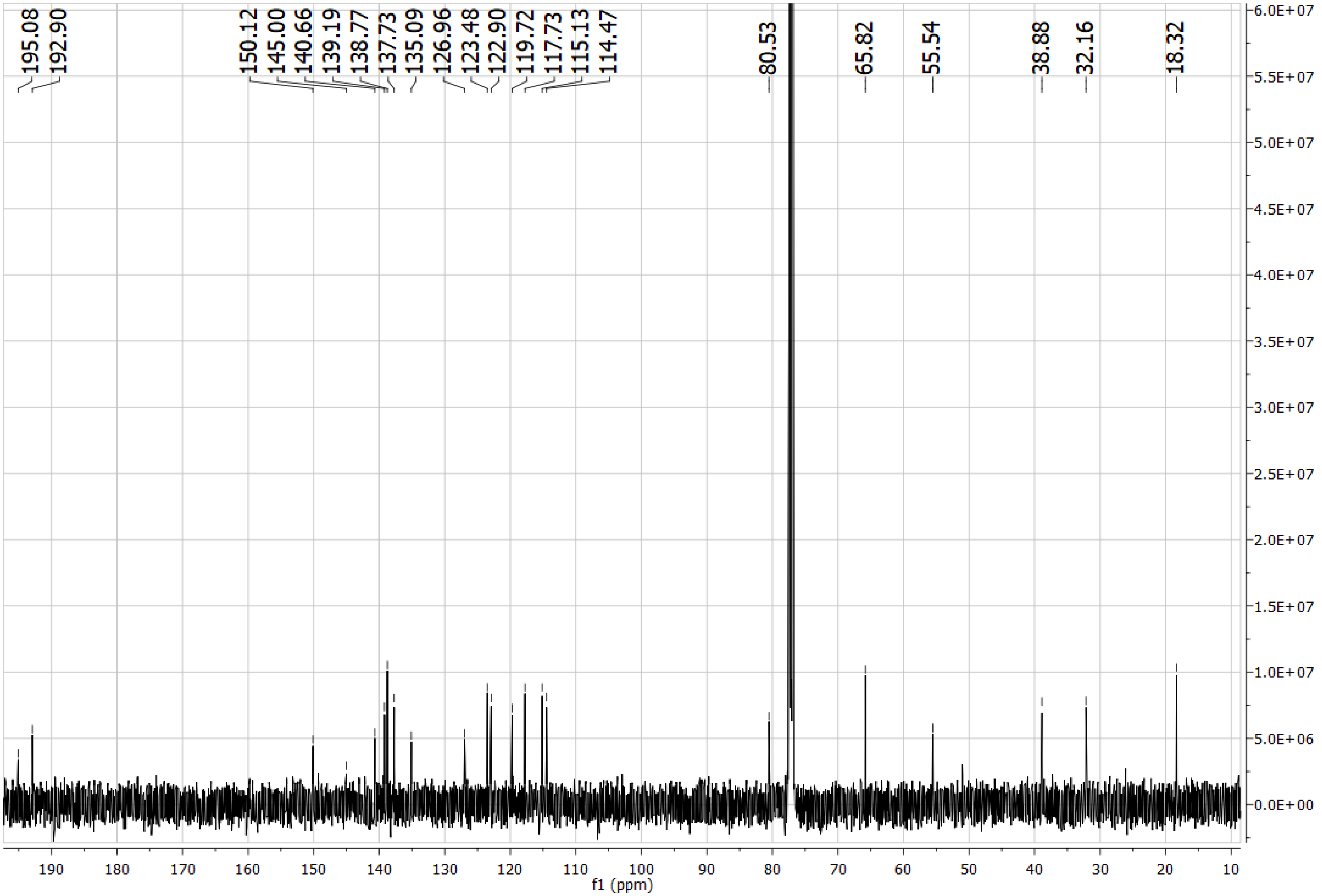

Figure S12. ^{13}C NMR spectrum of ehretiquinone C (**4**) measured in $CDCl_3$.

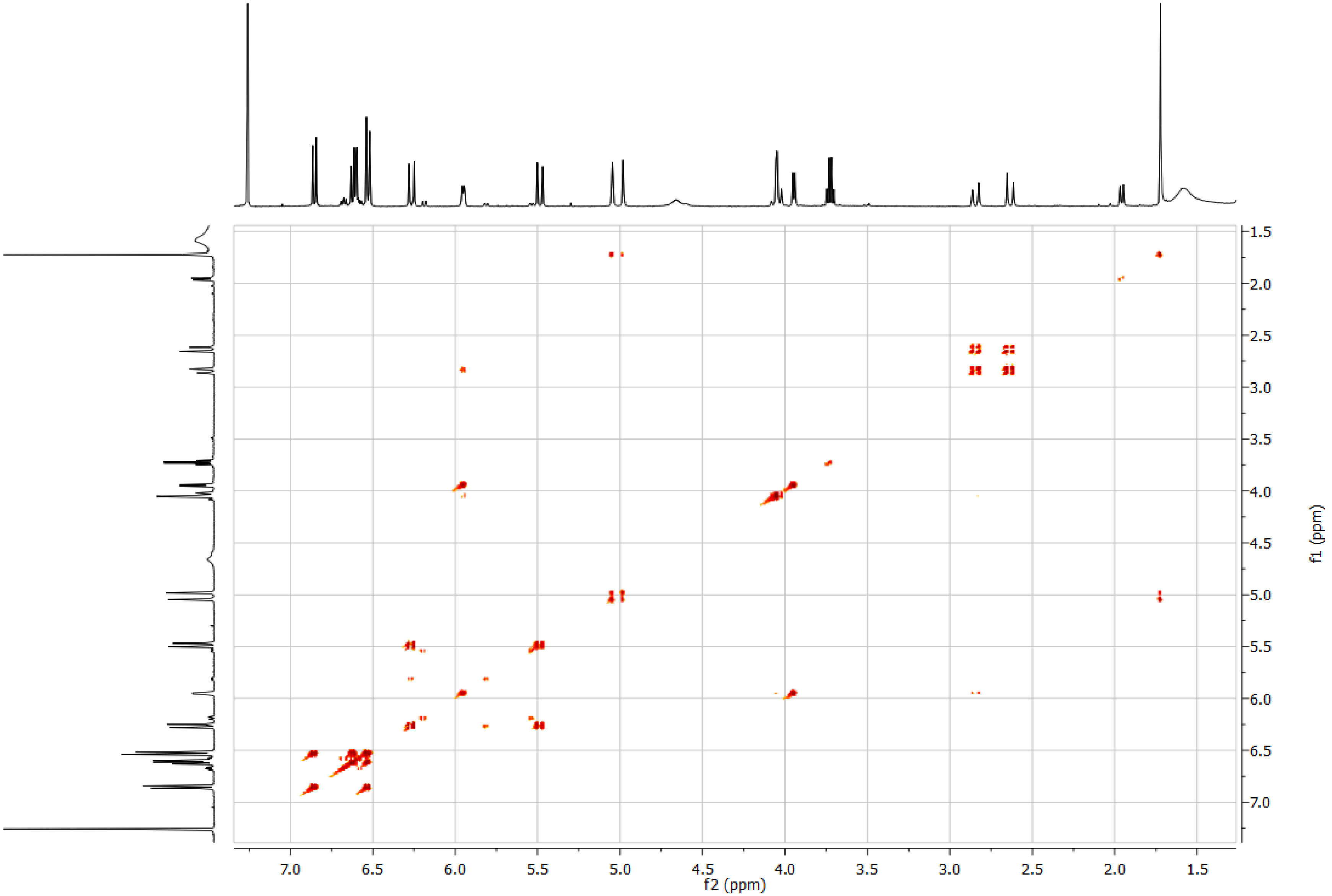

Figure S13. ^{1}H-^{1}H COSY spectrum of ehretiquinone C (**4**) measured in $CDCl_3$.

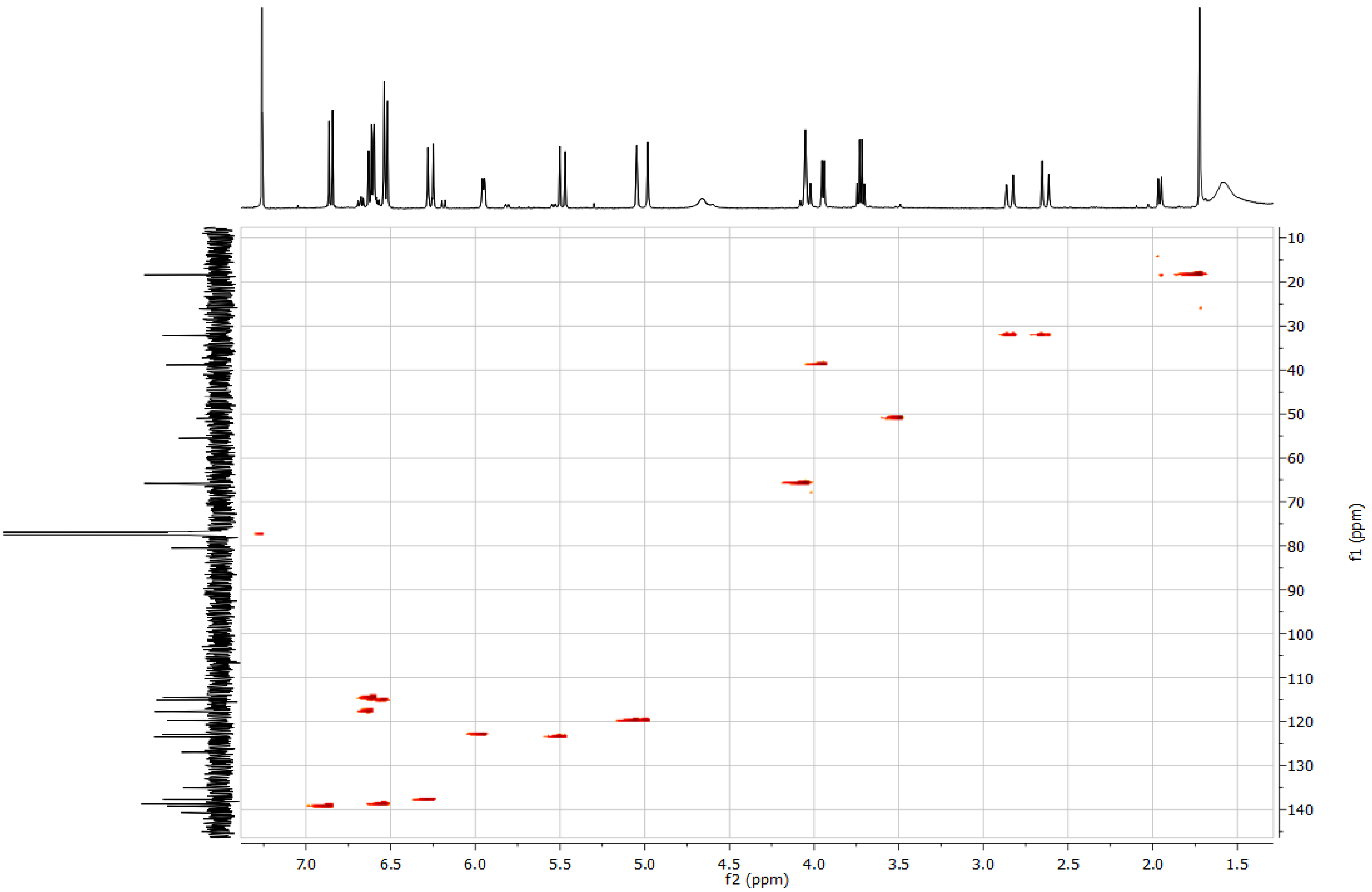

Figure S14. HSQC spectrum of ehretiquinone C (**4**) measured in $CDCl_3$.

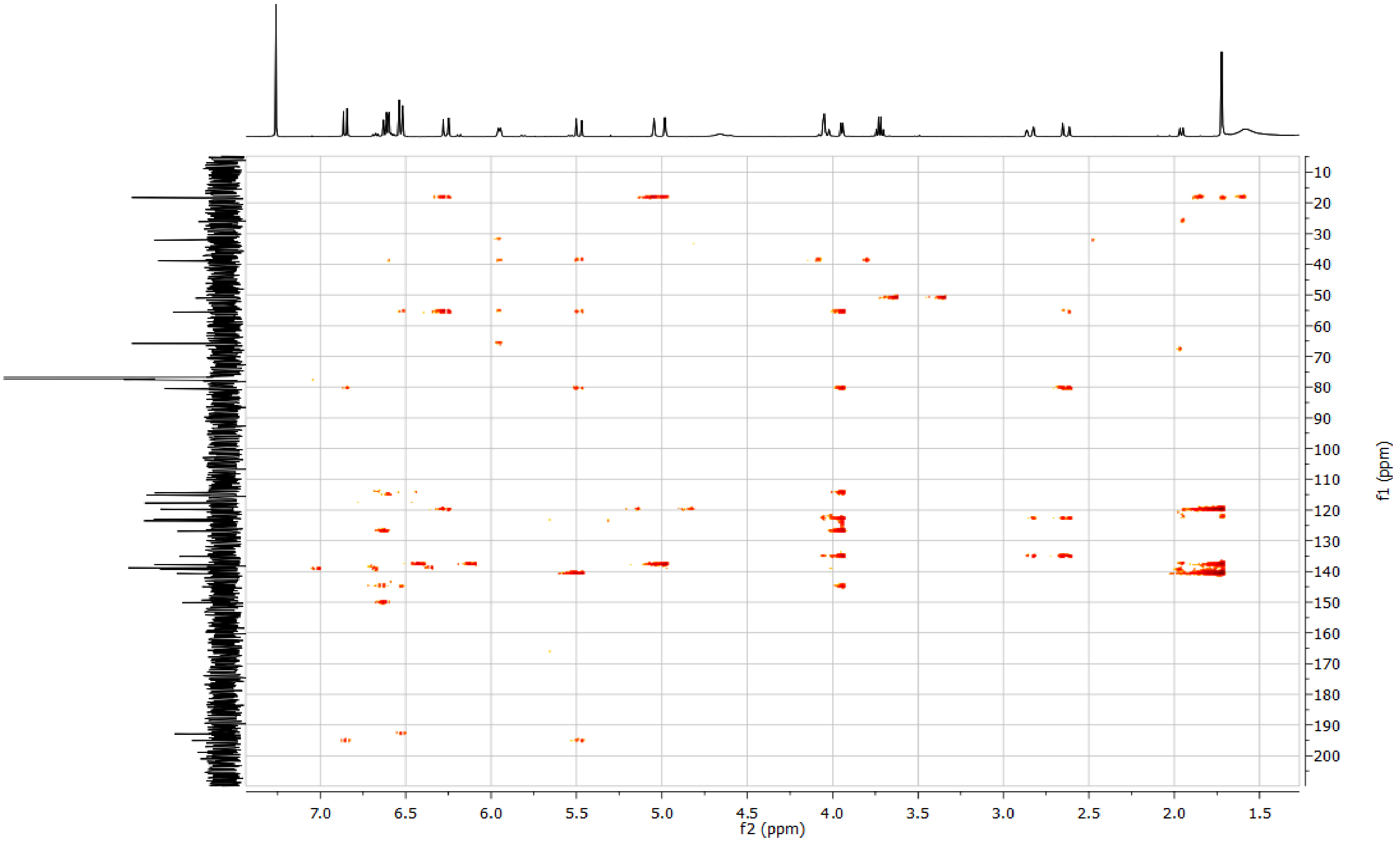

Figure S15. HMBC spectrum of ehretiquinone C (**4**) measured in $CDCl_3$.

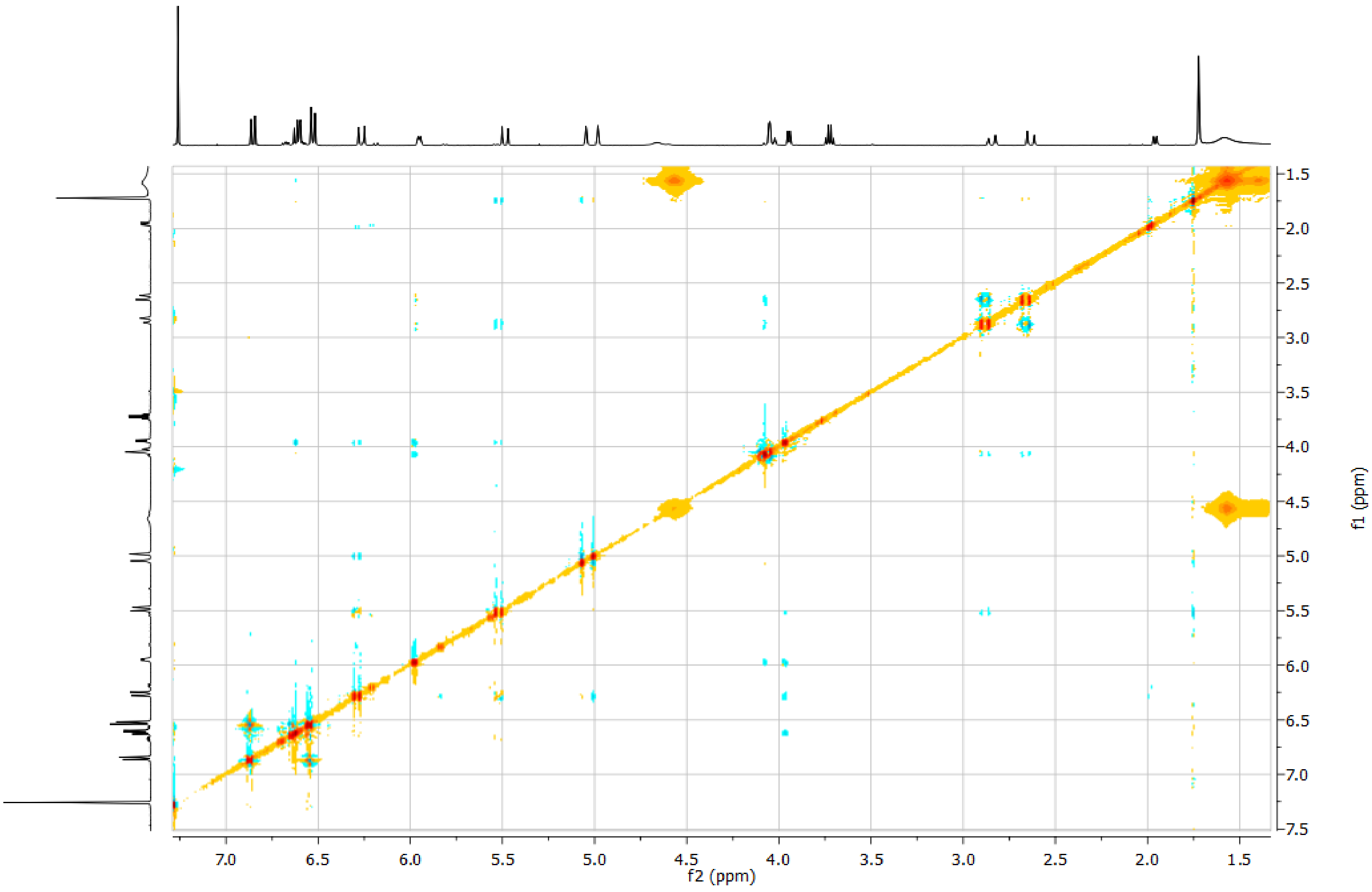

Figure S16. NOESY spectrum of ehretiquinone C (**4**) measured in $CDCl_3$.

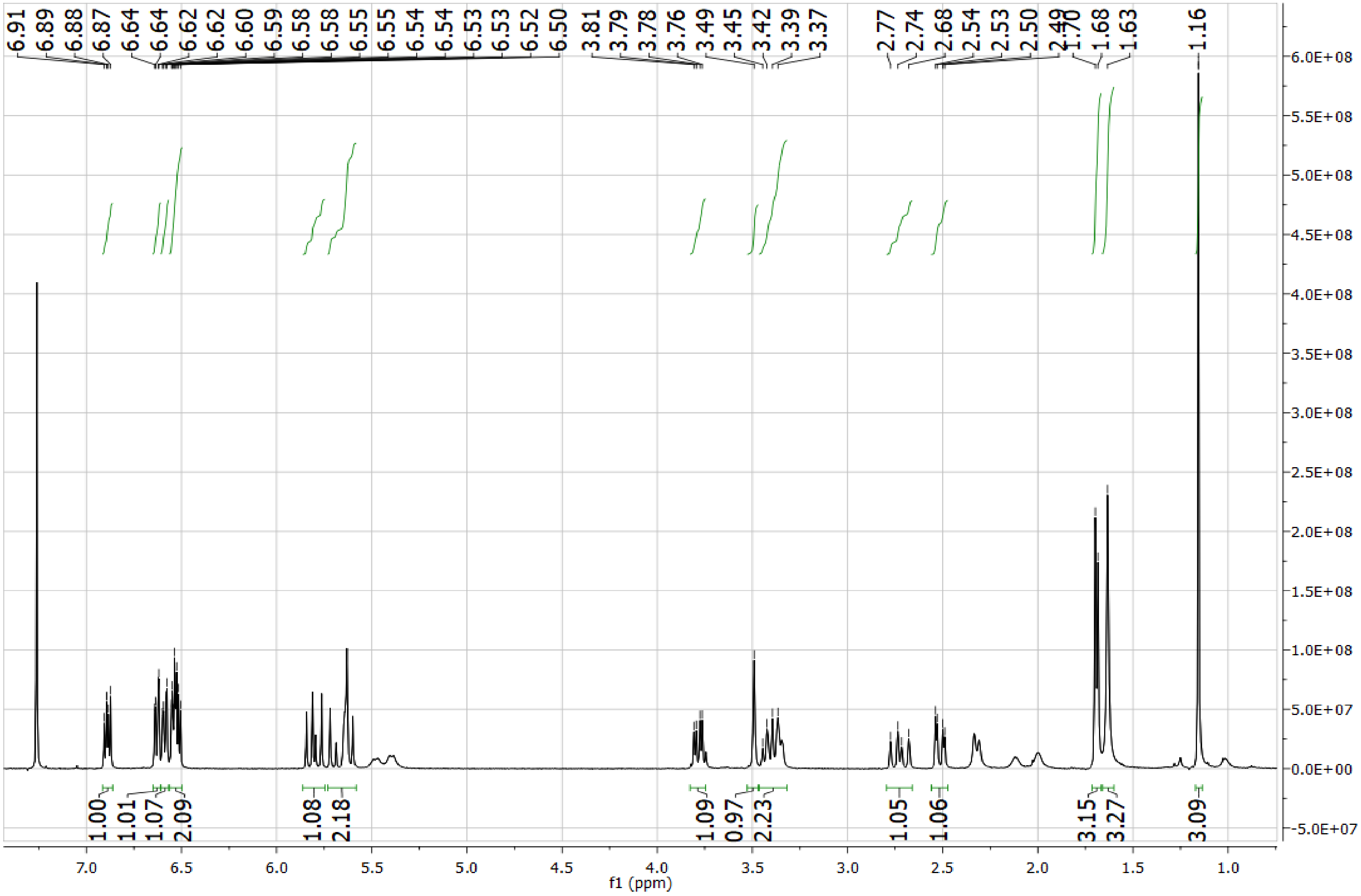

Figure S17. ^{1}H NMR spectrum of ehretiquinone D (**5**) measured in $CDCl_3$.

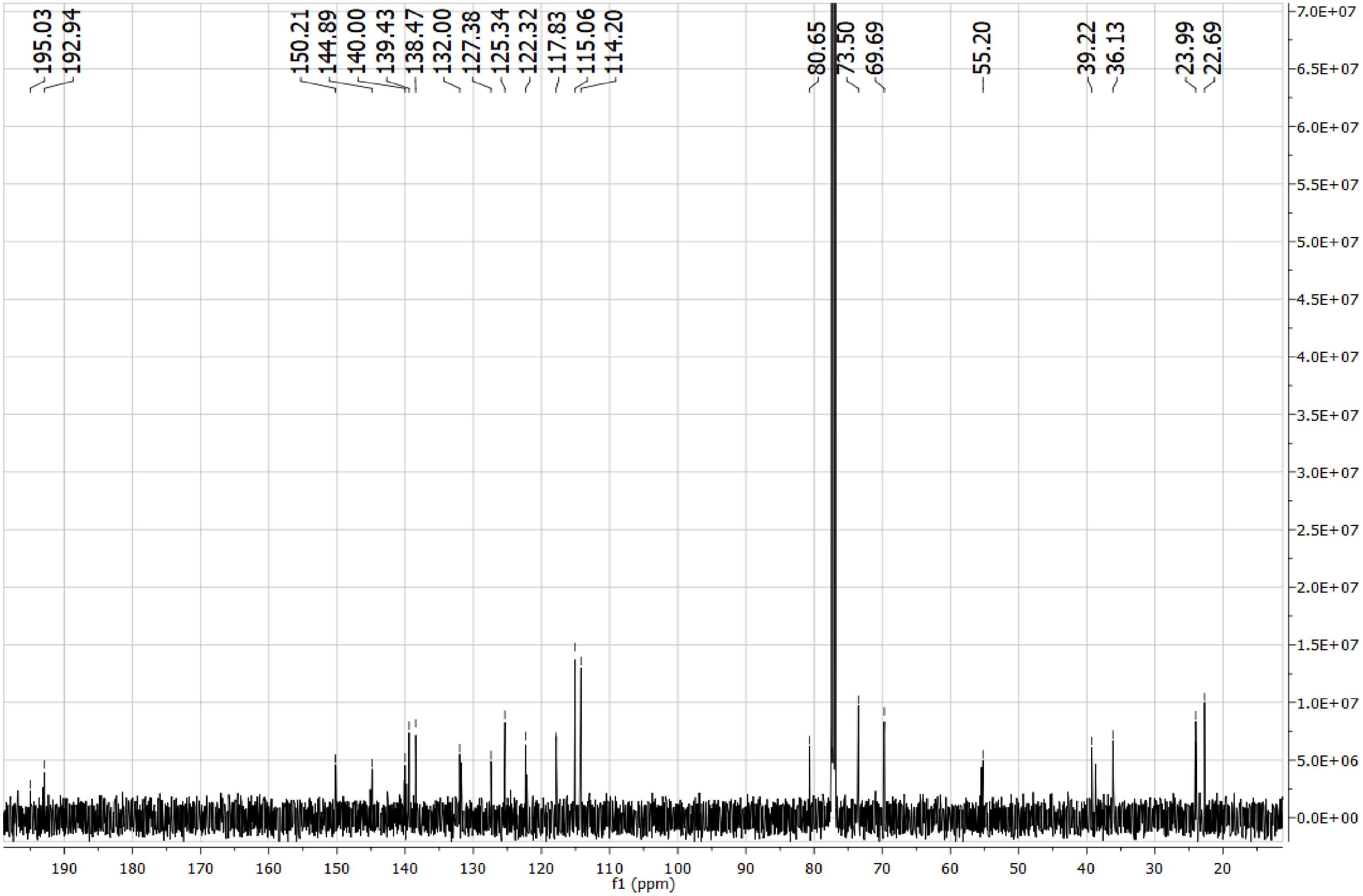

Figure S18. ^{13}C NMR spectrum of ehretiquinone D (**5**) measured in $CDCl_3$.

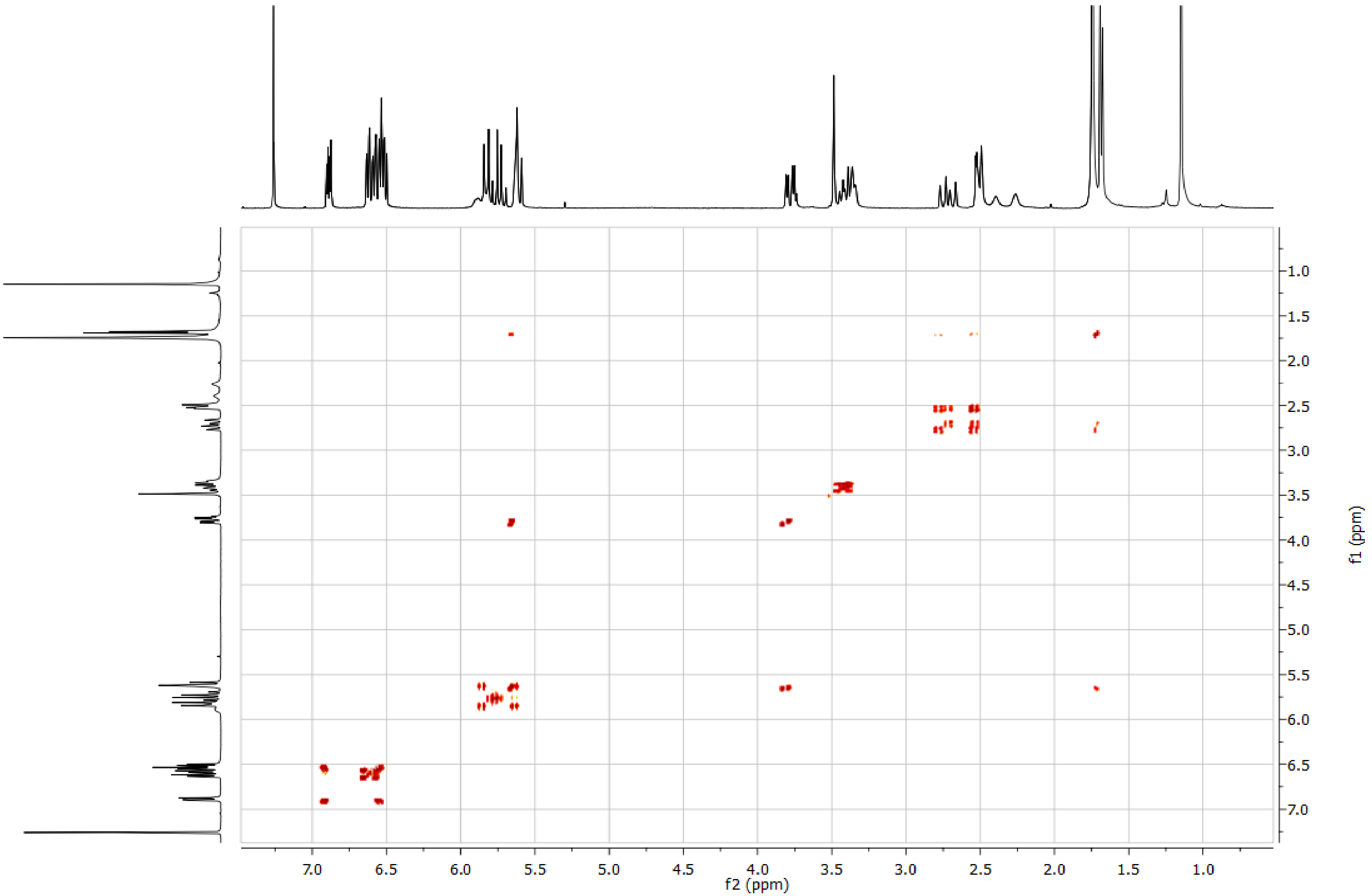

Figure S19. ^{1}H-^{1}H COSY spectrum of ehretiquinone D (**5**) measured in $CDCl_3$.

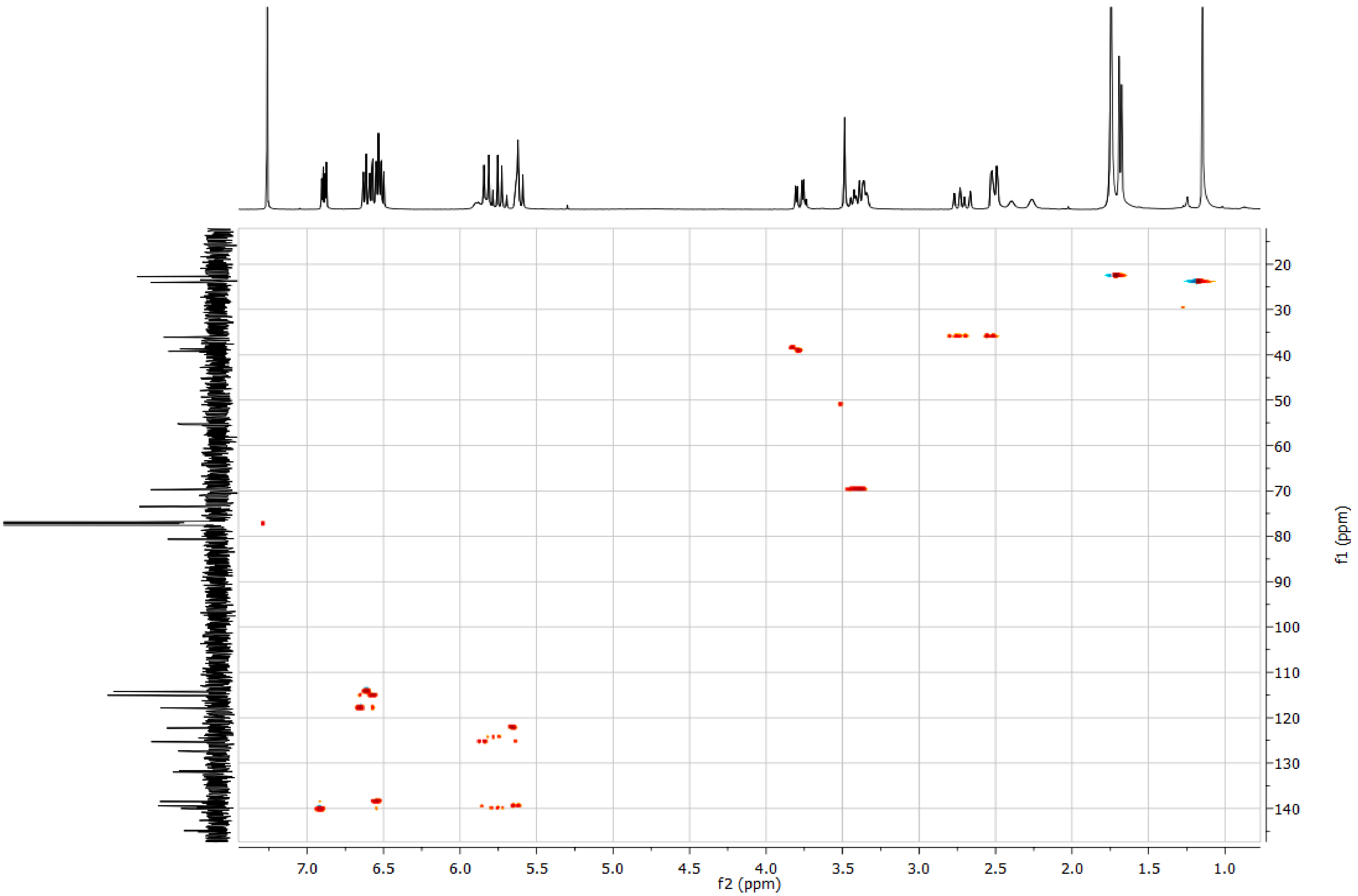

Figure S20. HSQC spectrum of ehretiquinone D (**5**) measured in $CDCl_3$.

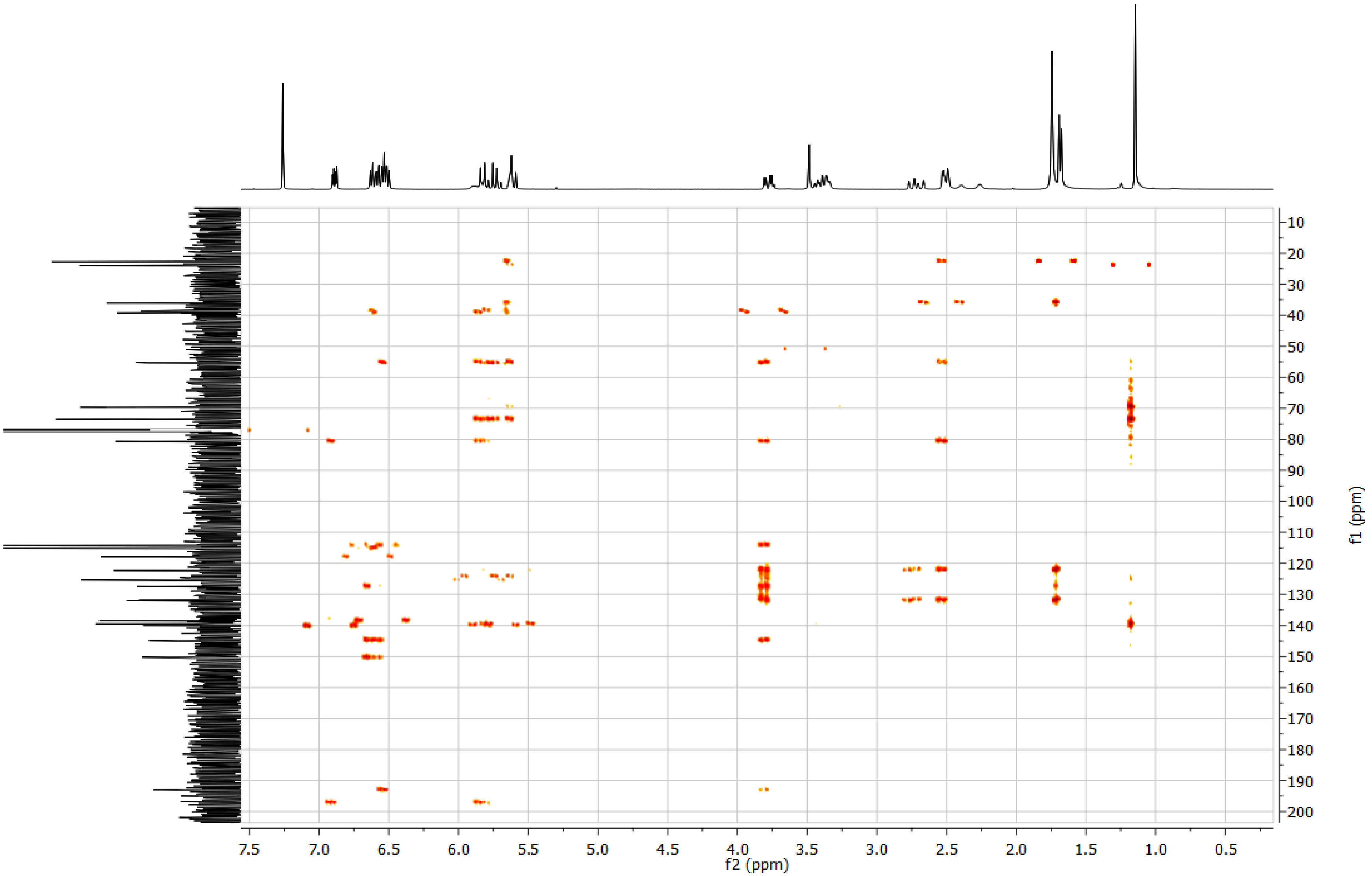

Figure S21. HMBC spectrum of ehretiquinone D (**5**) measured in $CDCl_3$.

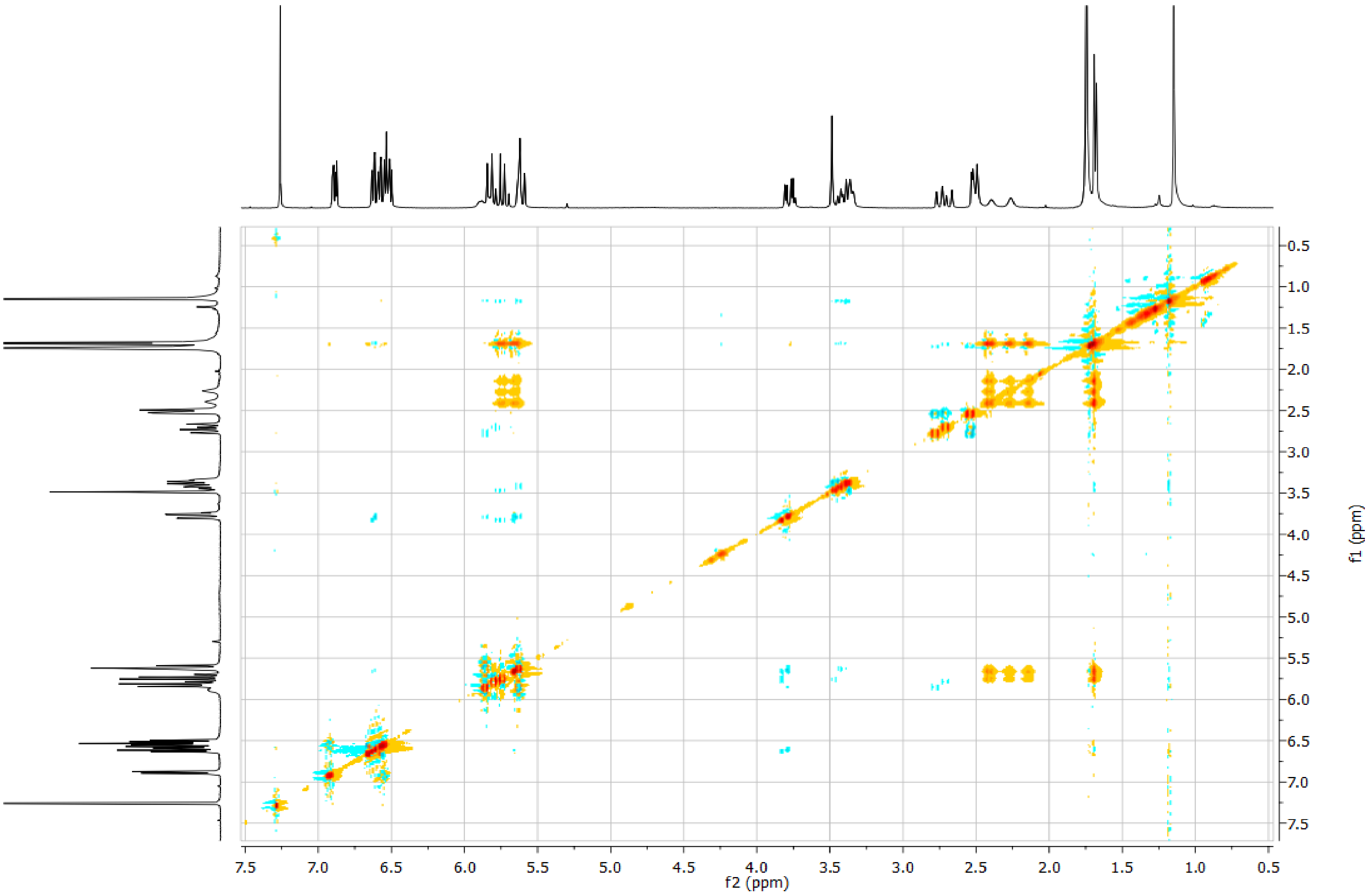

Figure S22. NOESY spectrum of ehretiquinone D (**5**) measured in $CDCl_3$.
